# Subjective aging and psychological well-being in older adults: a narrative review and multilevel framework integrating perceptual, behavioral, and sociocultural pathways

**DOI:** 10.3389/fpsyg.2026.1911901

**Published:** 2026-08-12

**Authors:** Siyao Song, Kaiyang Jia, Bowen Hou

**Affiliations:** School of Humanities and Social Sciences, Harbin Engineering University, Harbin, China

**Keywords:** ageism, awareness of age-related change, older adults, psychological well-being, self-perceptions of aging, subjective age, subjective aging

## Abstract

As the global population ages, understanding the psychological determinants of well-being in later life has become an important public health priority. A growing body of evidence suggests that how older adults experience, interpret, and evaluate their own aging—the domain of subjective aging—shapes psychological well-being. However, this literature remains fragmented across social gerontology, health psychology, and cultural psychology, with limited integration of perceptual, behavioral, and sociocultural mechanisms. This narrative review synthesizes recent and foundational literature on subjective aging and proposes a multilevel framework linking subjective aging constructs to psychological well-being. The framework identifies three interacting pathways: a perceptual pathway involving stereotype embodiment, stereotype threat, meaning-making, and age-related self-representations; a behavioral pathway through which evaluative self-perceptions of aging and awareness of age-related change influence health behaviors, social engagement, and goal-directed action; and a sociocultural pathway through which ageism, cultural norms, intergenerational relations, and socioeconomic conditions shape subjective aging. Evidence indicates that positive self-perceptions of aging and related appraisals are associated with lower depressive symptoms, greater life satisfaction, reduced loneliness, and longer survival, whereas negative appraisals predict psychological distress and social withdrawal. Implications for intervention, policy, and future research are discussed.

## 1 Introduction: subjective aging and psychological well-being

### 1.1 The global challenge of psychological well-being in aging populations

Population aging represents one of the defining demographic transformations of the 21st century. By 2050, the global population aged 60 years and over is projected to reach 2.1 billion, more than doubling from 1 billion in 2020 (World Health Organization, 2021a). This demographic shift brings a parallel increase in the burden of late-life mental health conditions: approximately 14.1% of adults aged 70 and over live with a mental disorder, with depressive and anxiety disorders among the leading contributors (World Health Organization, 2021a). A recent global burden analysis documented 74.9 million new mental disorder cases among older adults in 2021 alone (Chen et al., 2025). Beyond clinical disorders, subclinical psychological distress—including loneliness, diminished life satisfaction, and loss of purpose—affects a substantially larger proportion of the aging population and has been linked to functional decline and premature mortality (Holt-Lunstad et al., 2015).

Traditional mental health interventions face significant barriers in reaching older adults. Cognitive behavioral therapy, while efficacious, reaches only a minority due to workforce shortages, cost, mobility limitations, and ageist assumptions that psychological distress is a “normal” part of aging (Chisholm et al., 2016; Barbui et al., 2025). These challenges have spurred a shift toward identifying modifiable psychological resources that can serve as intervention targets before clinical thresholds are reached.

### 1.2 From deficit models to positive aging frameworks

For much of the twentieth century, gerontological research operated within a deficit paradigm emphasizing age-related decline, loss, and pathology (Baltes and Baltes, 1990). The emergence of positive aging frameworks—notably the successful aging model (Rowe and Kahn, 1997), the WHO active aging framework, and the UN Decade of Healthy Ageing (2021–2030)—has refocused attention on psychological resources that enable older adults to maintain well-being despite age-related challenges. Within this tradition, subjective aging has emerged as a broad field concerned with how individuals experience, interpret, and evaluate their own aging. In the present review, subjective aging is used as the superordinate term for this domain rather than as a single measurable construct.

Several related but non-equivalent constructs are distinguished within this domain. Self-perceptions of aging (SPA) refer to individuals’ beliefs, evaluations, and interpretations concerning their own aging (Diehl and Wahl, 2010). Attitudes toward own aging (ATOA) represent a specific evaluative assessment of one’s own aging experience (Lawton, 1975), whereas subjective age refers to how old individuals feel relative to their chronological age (Montepare, 2009). Awareness of age-related change (AARC) captures perceived gains and losses that individuals attribute to growing older across multiple life domains (Diehl and Wahl, 2010), while age identity concerns individuals’ identification with age-based social categories (Diehl et al., 2015). Although these constructs all concern the subjective experience of aging, they differ in conceptual content, measurement, and proposed mechanisms. Accordingly, the present review synthesizes them under the broader domain of subjective aging while retaining construct-specific terminology when reporting and interpreting empirical findings.

The foundational theoretical insight—articulated most prominently in Levy’s (2009) stereotype embodiment theory—is that societal age stereotypes are internalized across the lifespan and, when they become self-relevant in later life, exert profound influences on health, cognition, behavior, and well-being. Positive SPA and related subjective aging appraisals have been linked to a 7.5-year survival advantage (Levy et al., 2002), reduced risk of cognitive impairment (Siebert et al., 2018), greater engagement in preventive health behaviors (Levy and Myers, 2004), and enhanced psychological well-being (Wurm and Schäfer, 2022). Conversely, negative SPA and related appraisals predict accelerated functional decline, greater depressive symptoms, and increased all-cause mortality (Westerhof et al., 2023).

### 1.3 Conceptual positioning of the review

Despite compelling evidence, research on subjective aging and psychological well-being remains fragmented across sub-disciplines and construct traditions. Social gerontologists have focused on age identity and subjective age as aspects of the aging self (Diehl et al., 2015); health psychologists have investigated SPA as predictors of health behaviors and outcomes (Wurm et al., 2017); and sociologists have examined ageism as a structural determinant of late-life inequality (Chang et al., 2020). Accordingly, the central domain of this review is subjective aging rather than SPA alone. SPA is treated as a major evaluative construct within this domain, alongside subjective age, AARC, and age identity. Evidence is synthesized at the broader domain level only when findings converge across constructs; otherwise, the specific construct examined in the original study is retained. This review proposes that a comprehensive understanding requires integrating these streams into a unified multilevel framework with three interacting pathways:

#### 1.3.1 The perceptual pathway

In the present framework, “perceptual” does not refer narrowly to sensory perception. Rather, it denotes intrapersonal appraisal and self-representational processes through which age-related cues acquire personal meaning. Societal age stereotypes may shape evaluative SPA and other subjective aging experiences when they become self-relevant through internalization (Levy, 2009). Stereotype embodiment is included in this pathway because it describes the chronic process through which culturally available age stereotypes are incorporated into individuals’ self-views, whereas age-based stereotype threat captures the acute and situational activation of such stereotypes when individuals fear confirming them (Steele, 1997). Both processes operate by altering self-evaluations, emotional responses, interpretations of age-related experiences, and future expectations before or alongside overt action; they are therefore classified as perceptual rather than behavioral mechanisms. Subjective age is treated here as a distinct self-representation reflecting felt age relative to chronological age, rather than as a global perceptual summary or proxy for stereotype embodiment processes (Pinquart and Wahl, 2021).

#### 1.3.2 The behavioral pathway

The behavioral pathway concerns the observable actions through which subjective aging appraisals are enacted. Subjective aging constructs—particularly evaluative SPA and AARC—shape health-relevant behaviors, including physical activity, preventive healthcare utilization, and social engagement (Wurm et al., 2010; Levy and Myers, 2004). These behaviors mediate the association between SPA and psychological well-being: positive SPA promote active lifestyles and social participation, which independently reduce depressive symptoms and enhance life satisfaction, whereas negative SPA predict social withdrawal, creating a downward spiral of functional decline and psychological distress. Thus, this pathway is distinguished from the perceptual pathway by its focus on what individuals do rather than on how they internally interpret age-related information.

#### 1.3.3 The sociocultural pathway

The sociocultural pathway concerns the external relational, cultural, institutional, and socioeconomic conditions that generate, distribute, and reinforce meanings about aging. Subjective aging constructs are embedded within contexts shaped by ageism, cultural norms about aging, intergenerational relationships, and socioeconomic conditions (Kang and Kim, 2022; Schönstein et al., 2023). Age discrimination and structural ageism in healthcare, employment, and media representation contribute to negative SPA, while cultures that value older adults and promote intergenerational integration foster more positive views (Löckenhoff et al., 2009). Unlike the perceptual pathway, this pathway refers primarily to the social sources and structural reinforcement of age-related meanings rather than to their internal psychological processing.

**Figure 1:**
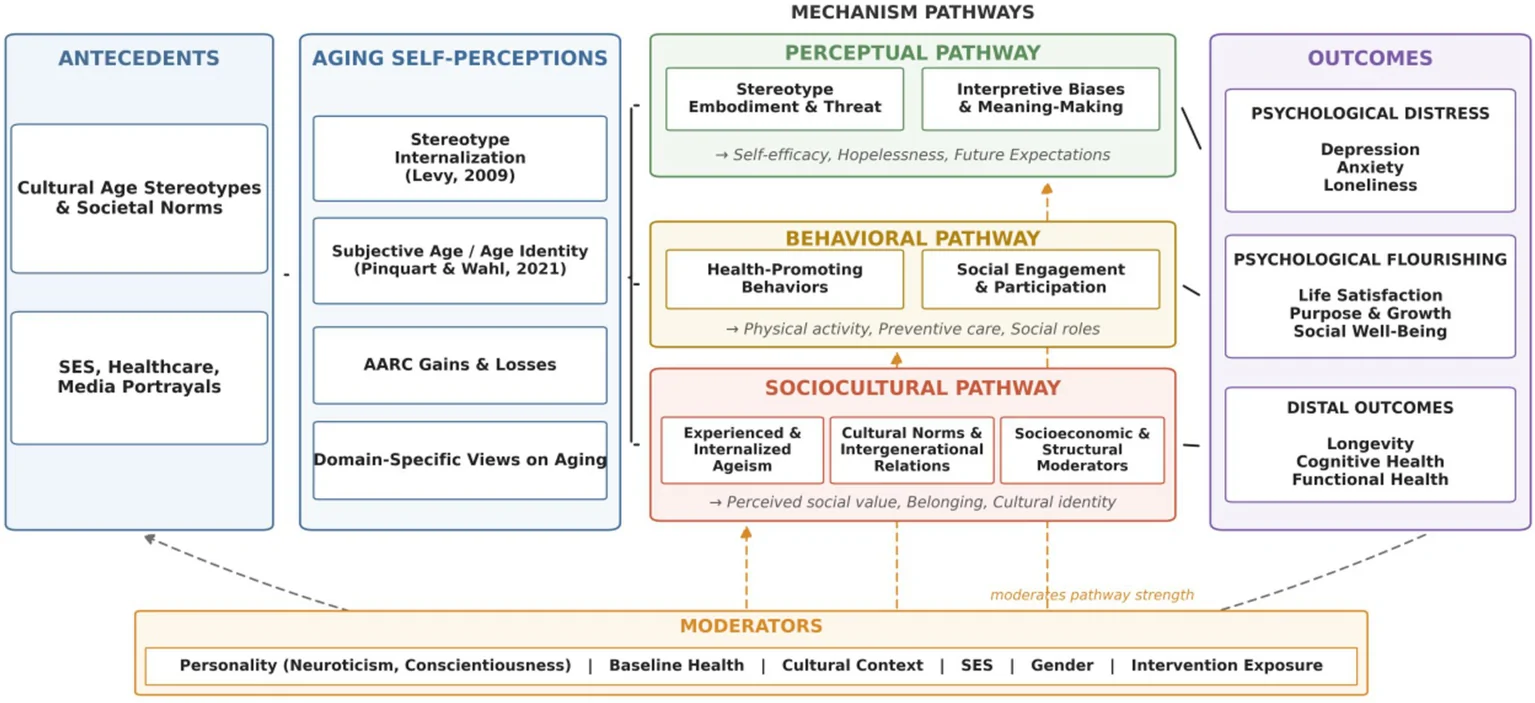
A multilevel framework linking aging self-perceptions to psychological well-being. Cultural, socioeconomic, healthcare, and media-related antecedents shape aging self-perceptions, which influence psychological distress, flourishing, and distal health outcomes through perceptual, behavioral, and sociocultural pathways. These relationships are moderated by individual and contextual factors, while feedback from outcomes may further reshape aging self-perceptions and broader societal views.

The three pathways are distinguished by their primary analytical locus and function. The perceptual pathway concerns how age-related information is internally appraised, interpreted, and incorporated into the aging self; the behavioral pathway concerns the observable actions through which these appraisals are enacted; and the sociocultural pathway concerns the external cultural, relational, institutional, and structural conditions that supply, reinforce, or constrain age-related meanings. These pathways are analytically distinguishable but dynamically interdependent: sociocultural conditions provide the content of age stereotypes, perceptual processes translate that content into self-relevant meanings, and behavioral responses enact and may subsequently reinforce those meanings over time. No single pathway alone is sufficient; a comprehensive model acknowledging their interdependence offers a more complete and clinically useful account. Taken together, these three pathways form the conceptual basis of the present review. Figure 1 provides an overview of the proposed multilevel framework, showing how sociocultural antecedents, subjective aging constructs, perceptual, behavioral, and sociocultural mechanisms, and psychological well-being outcomes are connected within a dynamic system.

### 1.4 Methodological approach

This narrative review synthesizes existing literature and proposes a novel multilevel conceptual model. We conducted a narrative synthesis emphasizing recent literature published between 2021 and 2026, while also including foundational theoretical and empirical publications necessary for conceptual and historical context. The databases searched were PubMed, PsycINFO, Web of Science, and Scopus. The search strategy comprised three concept blocks. The first block covered subjective aging and related age-stereotype concepts and included “aging self-perceptions,” “self-perceptions of aging,” “subjective aging,” “views on aging,” “age identity,” “subjective age,” “stereotype embodiment,” “ageism,” and “awareness of age-related change.” The second and third blocks consisted of “psychological well-being” and “older adults,” respectively. Terms within the first block were combined using the Boolean operator “OR,” and the three concept blocks were then combined using “AND.” The core search logic was therefore: (“aging self-perceptions” OR “self-perceptions of aging” OR “subjective aging” OR “views on aging” OR “age identity” OR “subjective age” OR “stereotype embodiment” OR “ageism” OR “awareness of age-related change”) AND “psychological well-being” AND “older adults.” The same conceptual search structure was applied across all four databases. Only the field tags and database-specific syntax required by each platform were adjusted, while the search concepts and Boolean structure remained unchanged. Inclusion criteria encompassed peer-reviewed English-language publications examining at least one subjective aging construct in relation to psychological well-being among adults aged 50 and older. During the narrative synthesis, studies were classified according to the construct actually assessed: evaluative SPA and related views-on-aging measures, subjective age or age identity, and AARC. We used the umbrella term subjective aging only when discussing patterns that extended across more than one construct and retained construct-specific terminology when reporting individual findings. The final body of literature included recent empirical studies, systematic reviews and meta-analyses, theoretical contributions, and foundational publications relevant to subjective aging and psychological well-being. Because of substantial heterogeneity in study designs, measures, populations, and outcomes, a formal meta-analysis was not conducted. Instead, the evidence was synthesized narratively and organized according to the perceptual, behavioral, and sociocultural pathways proposed in the present framework.

## 2 Conceptual framework: a multilevel model of subjective aging and psychological well-being

Subjective aging constructs can be conceptualized as components of a multilevel system in which individual appraisals, enacted behaviors, and sociocultural conditions interact dynamically. In this framework, the perceptual pathway comprises internalization, appraisal, meaning-making, and self-representational processes, including stereotype embodiment, stereotype threat, and the interpretation of subjective age and age identity; the behavioral pathway comprises observable responses such as health behaviors, social engagement, and goal pursuit; and the sociocultural pathway comprises external influences such as ageism, cultural norms, intergenerational relations, and socioeconomic conditions. Figure 1 illustrates the proposed framework.

Although this multilevel framework draws upon established theoretical models—including stereotype embodiment theory (Levy, 2009), the motivational theory of lifespan development (Heckhausen et al., 2010), the SOC model (Baltes and Baltes, 1990), and cultural-psychological perspectives on aging (Löckenhoff et al., 2009)—it provides a structured synthesis specifically tailored to explain how subjective aging constructs are formed, enacted, and linked to psychological well-being. Unlike prior models focusing on single mechanisms—such as stereotype threat effects on cognitive performance (Lamont et al., 2015) or age identity in isolation (Montepare, 2009)—the present model emphasizes the interplay of perceptual, behavioral, and sociocultural pathways within the developmental trajectory. Its novelty lies in synthesizing and extending existing theories into a domain-specific architecture that explains how evaluative, identity-related, awareness-based, and sociocultural dimensions of subjective aging are linked to psychological well-being.

### 2.1 Conceptual taxonomy of subjective aging constructs

The present framework distinguishes three broad families of subjective aging constructs: evaluative self-perceptions of aging, identity-related constructs such as subjective age and age identity, and awareness-based constructs represented by AARC. Age stereotypes, structural ageism, interpersonal ageism, and cultural norms are treated as sociocultural antecedents or contextual influences rather than as subjective aging constructs themselves. Internalized ageism, also referred to as self-directed ageism, occupies an intermediate position: it arises when socially transmitted ageist beliefs are accepted and applied to the self. It may therefore be understood as a specific negative manifestation of self-perceptions of aging, but it does not encompass the broader SPA construct, which also includes positive, neutral, multidimensional, and domain-specific evaluations of one’s own aging. Within the proposed framework, stereotype internalization and meaning-making are classified as perceptual mechanisms, health-related behavior and goal pursuit as behavioral mechanisms, and ageism and cultural norms as sociocultural influences; perceived control is treated as a self-regulatory bridge through which perceptual appraisals are translated into goal-directed behavior. Stereotype internalization represents the foundational process through which societal age stereotypes become self-relevant (Levy, 2009). From childhood, individuals are exposed to culturally transmitted beliefs about aging. These stereotypes remain latent through young adulthood but become increasingly accessible and self-relevant in midlife and beyond. The distinction between general (societal) and personal views of aging is critical: Brothers et al. (2021) demonstrated that the effects of age stereotypes on physical and mental health are fully mediated by personal self-perceptions of aging, supporting the proposition that internalization is the mechanism through which cultural-level phenomena manifest at the individual level.

Subjective age reflects how old an individual feels relative to their chronological age. Pinquart and Wahl (2021) found in their comprehensive meta-analysis that adults across the lifespan tend to feel approximately 20% younger than their chronological age from midlife onward, with the discrepancy increasing with advancing age. A younger subjective age is prospectively associated with better health outcomes, greater well-being, and lower mortality risk (Westerhof et al., 2023; Stephan et al., 2018), operating through lower physiological stress reactivity, reduced inflammation (Stephan et al., 2015), and greater engagement in health-promoting behaviors.

Attitudes toward own aging (ATOA) represent a unidimensional evaluative judgment of one’s personal aging experience, assessed via the Philadelphia Geriatric Center Morale Scale (Lawton, 1975). Longitudinal evidence from the ILSE study has shown that ATOA predicts cognitive decline, functional health, and mortality over 12 years (Siebert et al., 2018).

Awareness of age-related change (AARC) is a multidimensional construct distinguishing perceived gains and losses in five behavioral domains (Diehl and Wahl, 2010; Brothers et al., 2019). Wurm and Schäfer (2022) found that gain-related—but not loss-related—self-perceptions predicted mortality over 23 years, suggesting that the presence of positive SPA may be more consequential than the absence of negative ones for longevity.

Domain-specific views on aging (VoA) extend this logic by recognizing that individuals may hold different aging expectations across life domains (Kornadt and Rothermund, 2011), enabling more precise prediction of domain-congruent outcomes.

To clarify the conceptual boundaries among these related constructs, Table 1 summarizes the major subjective aging constructs, their associated mechanisms, contextual moderators, and psychological well-being outcomes. The table also distinguishes subjective aging constructs from sociocultural antecedents and cross-level processes such as internalized ageism.

**Table 1:** Key subjective aging constructs, related mechanisms, and contextual influences relevant to psychological well-being.

Level/category	Key component/feature	Description/primary effects	Moderators/design influences	Associated mediating processes	Key outcomes/psychological well-being effects	Reference(s)
Perceptual/Evaluative	Stereotype internalization	Societal age stereotypes internalized across lifespan; become self-relevant in old age; operate through implicit (automatic priming) and explicit (interpretive biases) processes	Trait absorption, age identification, baseline self-concept clarity	Interpretive bias, stereotype threat/boost, self-fulfilling prophecy processes	Negative internalization predicts ↑ depressive symptoms, ↓ life satisfaction; positive internalization linked to 7.5-year survival advantage	Levy (2009, 2022), Brothers et al. (2021), Weiss and Kornadt (2018)
Perceptual/Evaluative	Subjective age (felt age)	How old one feels relative to chronological age; adults feel ~20% younger from midlife onward; discrepancy increases with age	Chronological age, health status, age discrimination experiences, cultural aging norms	Optimism, self-efficacy, future time perspective expansion; lower CRP, reduced autonomic stress reactivity	Younger subjective age predicts ↓ depressive symptoms, ↓ anxiety, ↓ mortality; d ≈ 0.30–0.50 across meta-analyses	Pinquart and Wahl (2021), Westerhof et al. (2023), Stephan et al. (2015, 2018)
Perceptual/Evaluative	Attitudes toward own aging (ATOA)	Unidimensional evaluative judgment of personal aging; assessed via 5-item Philadelphia Geriatric Center Morale Scale subscale	Gender (women report more negative ATOA), baseline health, SES, marital transitions	Perceived control, hopelessness, cognitive appraisals of age-related changes	Negative ATOA predicts 3 × greater hippocampal volume loss, ↑ MCI/Alzheimer’s risk over 12 years; predicts depressive symptoms	Lawton (1975), Siebert et al. (2018), Levy et al. (2016), Turner et al. (2023)
Awareness-Based/Multidimensional Gain–Loss	Awareness of age-related gains (AARC-Gains)	Perceived positive changes that individuals explicitly attribute to growing older across five domains: health and physical functioning, cognitive functioning, interpersonal relations, social-cognitive and socioemotional functioning, and lifestyle and engagement.	Personality traits, cultural context, health status, social resources, and life-stage conditions	Tenacious goal pursuit, perceived control, intrinsic motivation, meaning-making, and continued social engagement	Higher AARC-Gains are associated with greater psychological well-being, more positive affect, stronger goal engagement, and lower mortality risk.	Diehl and Wahl (2010), Brothers et al. (2019), Wurm and Schäfer (2022), Kaspar et al. (2019)
Awareness-Based/Multidimensional Gain–Loss	Awareness of age-related losses (AARC-Losses)	Perceived negative changes that individuals explicitly attribute to growing older across the same five life domains assessed by the AARC framework.	Objective health decline, negative life events, socioeconomic disadvantage, cultural context, and available coping resources	Reduced perceived control, flexible goal adjustment, secondary control, compensatory strategy use, and social withdrawal	Higher AARC-Losses are associated with greater depressive symptoms and negative affect, lower life satisfaction and self-rated health, and reduced psychological well-being.	Diehl and Wahl (2010), Brothers et al. (2019), Dutt et al. (2018), Schönstein et al. (2023)
Domain-Specific/Contextual	Domain-specific views on aging (VoA)	Differentiated expectations about aging across life domains (family, work, health, leisure, cognitive fitness); enables precise, domain-congruent prediction	Domain salience, personal experience in domain, cultural role expectations	Domain-specific behavioral engagement (e.g., physical activity, cognitive training, social participation)	Domain-congruent outcomes: physical VoA predicts physical activity; cognitive VoA predicts cognitive engagement; social VoA predicts social participation	Kornadt and Rothermund (2011, 2015), Wurm et al. (2017)
Behavioral/Self-Regulatory	Perceived control over aging	Domain-specific belief that one can influence the trajectory of one’s own aging; distinct from general locus of control	Education, health literacy, prior mastery experiences, age stereotypes	Primary control (tenacious goal pursuit), secondary control (goal adjustment), SOC strategy use	Higher control predicts ↓ depressive symptoms, ↑ physical activity, better functional health; mediates SPA–well-being link	Lachman (2006), Heckhausen et al. (2010), Zhang and Neupert (2021), Dutt et al. (2018)
Cross-Level/Sociocultural-to-Perceptual	Internalized ageism (self-directed ageism)	Acceptance and application of negative age stereotypes to self-evaluation; represents convergence of sociocultural and perceptual pathways	Experienced age discrimination, media exposure to negative aging portrayals, healthcare age bias	Reduced health behaviors, social withdrawal, diminished healthcare seeking	Predicts ↑ depressive symptoms, ↑ anxiety, ↑ loneliness; interacts with other forms of marginalization (syndemic effects)	Kang and Kim (2022), Chang et al. (2020), Shen et al. (2025),
Sociocultural/Structural	Cultural aging norms	Culturally transmitted expectations about the aging process and older adults’ roles; shape content and valence of aging self-perceptions	Cultural values (individualism vs. collectivism), filial piety norms, welfare state regime, economic development	Cultural moderation of objective health–SPA link; meaning-making and identity affirmation processes	Cross-cultural variation: higher SPA in cultures with multigenerational households and elder-valued traditions (e.g., Burkina Faso vs. Germany)	Löckenhoff et al. (2009), Schönstein et al. (2023), Osawa et al. (2024)

### 2.2 Mediating processes

#### 2.2.1 Perceptual mediation: stereotype embodiment, situational appraisal, and meaning-making

Perceptual mediation refers to changes in psychological well-being that occur because age-related information alters how individuals interpret themselves, their experiences, and their future prospects, rather than because it directly changes their behavior or external social context. The primary developmental mechanism is stereotype embodiment (Levy, 2009, 2022). When individuals encounter age-related changes, those who have internalized negative age stereotypes are more likely to interpret these experiences as evidence of irreversible decline, triggering helplessness and diminished self-efficacy (Weiss and Kornadt, 2018). Conversely, those with positive SPA may interpret the same experiences as normative and manageable. Age-based stereotype threat—the fear of confirming negative stereotypes about one’s age group—represents an acute and situational form of perceptual appraisal that can impair cognitive performance and increase physiological stress reactivity (Lamont et al., 2015). Although stereotype threat may subsequently produce behavioral or physiological consequences, it is classified within the perceptual pathway because its initiating mechanism is the self-relevant appraisal of an age-salient situation.

#### 2.2.2 Behavioral mediation: health behaviors and social engagement

Behavioral mediation begins when subjective aging appraisals are translated into observable health-related, social, or goal-directed actions. Older adults with more positive SPA are more likely to engage in physical activity, adhere to preventive healthcare, maintain healthy diets, and avoid health-risk behaviors (Levy and Myers, 2004; Wurm et al., 2010). SPA also influence self-care behaviors in older adults with chronic conditions (Mezza et al., 2025). Social engagement represents a second behavioral mechanism: positive SPA predict greater social participation and larger, more diverse social networks (Cai et al., 2024). Conversely, negative SPA predict social withdrawal, creating a vicious cycle whereby isolation reinforces negative self-views (Kang and Kim, 2022). Unlike perceptual mediation, which operates through changes in appraisal and self-representation, behavioral mediation operates through changes in what individuals actually do.

#### 2.2.3 Goal regulatory mediation: control beliefs and adaptive coping

According to the motivational theory of lifespan development (Heckhausen et al., 2010), successful aging requires flexible use of primary and secondary control strategies. Perceived control over aging—the belief that one can influence the trajectory of one’s own aging—predicts greater use of adaptive coping (Lachman, 2006). Dutt et al. (2018) found that awareness of age-related gains was associated with tenacious goal pursuit, whereas awareness of losses was associated with flexible goal adjustment. Zhang and Neupert (2021), using ecological momentary assessment, demonstrated that within-person fluctuations in control beliefs mediated the daily association between awareness of aging and affect.

**Figure 2:**
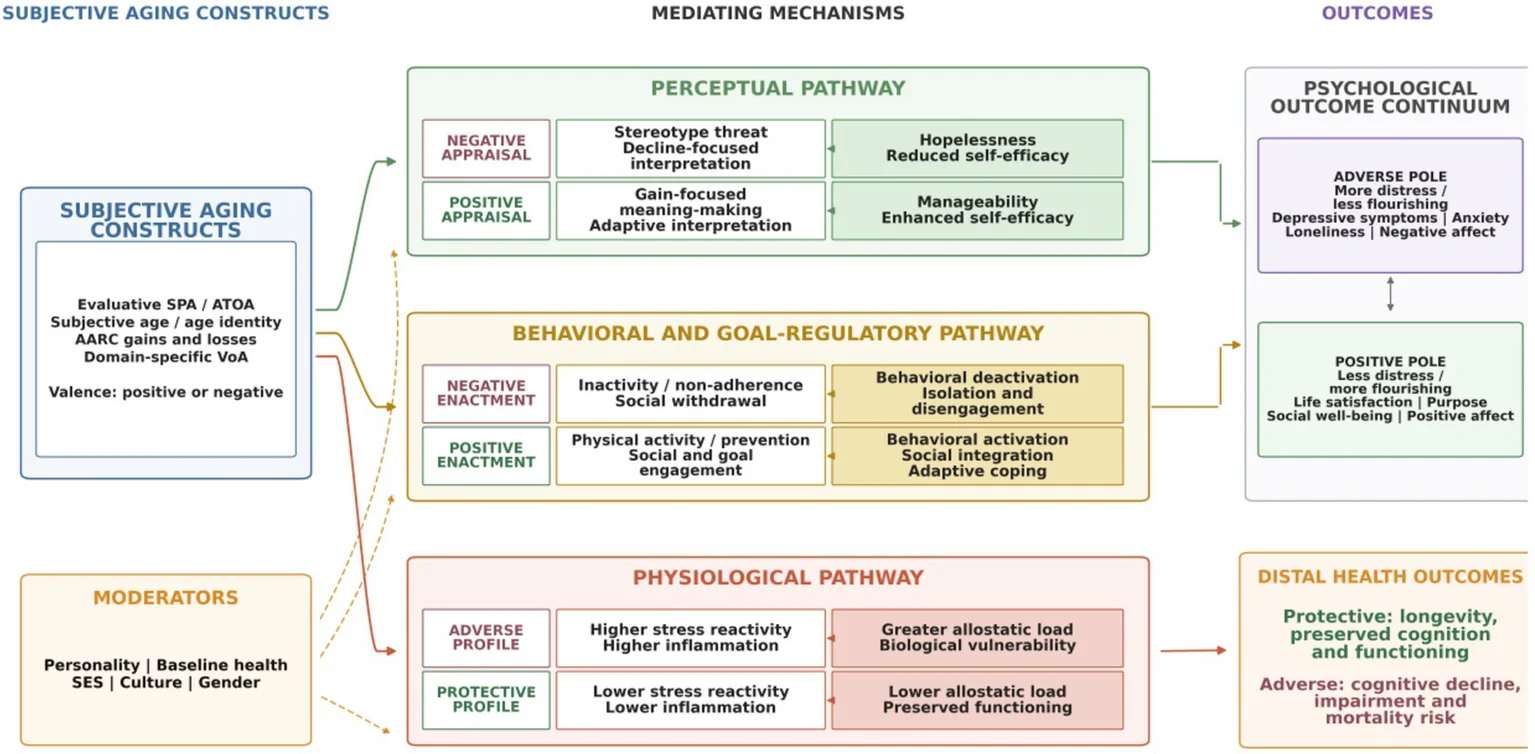
Bidirectional mechanisms linking subjective aging constructs to psychological well-being. Subjective aging constructs influence psychological well-being through perceptual, behavioral and goal-regulatory, and physiological pathways. Perceptual and behavioral processes may operate in both positive and negative directions, while physiological processes are linked primarily to distal health outcomes. Moderators may alter the strength of these pathways.

Taken together, these mediating processes clarify how subjective aging appraisals are translated into psychological well-being outcomes. Figure 2 summarizes this mechanism-oriented model by distinguishing perceptual, behavioral and goal-regulatory, and physiological pathways, while also showing how positive and negative subjective aging appraisals may shift individuals toward different positions on the psychological outcome continuum.

### 2.3 Downstream outcomes

In the domain of psychological distress, the updated meta-analysis by Westerhof et al. (2023) of 107 longitudinal studies found that negative subjective aging significantly predicted increased depressive symptoms and anxiety, with small but consistent effect sizes. A 2025 meta-analysis of 33 studies found that more positive self-perceptions of aging were moderately associated with higher overall psychological well-being, including greater hedonic well-being and lower levels of mental ill-health, such as depression and anxiety (Ugwu and Idemudia, 2025).

In positive psychological functioning, older adults with positive SPA report greater sense of purpose, personal growth, better social relationships, and greater environmental mastery—all dimensions of Ryff’s (1989) psychological well-being model. Bar-Tur (2021) argued that fostering positive aging self-perceptions should be a central goal of positive aging interventions.

Regarding longevity, Levy et al. (2002) demonstrated that older adults with more positive self-perceptions of aging lived 7.5 years longer than those with more negative self-perceptions. Wurm and Schäfer (2022) replicated this over 23 years, showing that gain-related SPA specifically predicted survival.

For social well-being, positive SPA predict greater social participation, higher perceived social support, and lower loneliness (Cai et al., 2024; Kang and Kim, 2022).

## 3 Perceptual and behavioral pathways

### 3.1 Stereotype embodiment and perceptual mechanisms

Levy’s (2009) stereotype embodiment theory proposes four key premises: stereotypes become internalized across the lifespan, operate unconsciously, gain salience from self-relevance, and utilize psychological, behavioral, and physiological pathways. The present framework does not equate all components of stereotype embodiment theory with the perceptual pathway. Instead, it classifies the internalization, activation, and interpretation of age stereotypes as perceptual processes; the resulting health-related and social actions as behavioral processes; and the cultural production and institutional reinforcement of age stereotypes as sociocultural processes. Brothers et al. (2021) provided the first formal test of the mediation hypothesis: using longitudinal data from the United States and Germany, they found that the effects of general age stereotypes on physical and mental health were mediated by personal awareness of age-related change, assessed using AARC (Brothers et al., 2021).

In the present framework, perceptual mechanisms include both automatic activation and controlled appraisal. Priming studies demonstrate that subliminal exposure to negative age stereotypes impairs memory performance, increases cardiovascular stress reactivity, and reduces walking speed in older adults (Levy, 2009). At the controlled level, explicit SPA influence the interpretive frames applied to age-related experiences. Weiss and Kornadt (2018) distinguished between internalization, through which individuals unconsciously assimilate stereotypes, and dissociation, through which they consciously distance themselves from their age group, proposing that both can operate simultaneously. Their common feature is that they determine how age-related information becomes self-relevant and is interpreted, which is the defining function of the perceptual pathway.

Age-based stereotype threat (Steele, 1997) represents the acute and situational form of this perceptual process. When age stereotypes become salient, individuals may appraise the situation as carrying a risk of confirming a negative stereotype about their age group, producing anticipatory distress and impaired performance. Lamont et al. (2015) demonstrated that stereotype threat effects are robust across multiple domains and may vary according to individuals’ subjective aging appraisals and age-related self-views. Although its consequences may be behavioral or physiological, stereotype threat is classified as perceptual because its initiating mechanism is the self-relevant interpretation of an age-salient situation. Key measurement instruments include the ATOA subscale (Lawton, 1975), the Aging Perceptions Questionnaire (Barker et al., 2007), and the AgeCog scales (Steverink et al., 2001).

### 3.2 Subjective age and age identity

Subjective age is the most extensively studied construct in the subjective aging literature. Although subjective age is associated with evaluative SPA, the two constructs are not interchangeable: subjective age primarily captures felt-age discrepancy or age identification, whereas SPA capture evaluations and beliefs concerning one’s own aging. Pinquart and Wahl (2021) found that adults feel approximately 20% younger than their chronological age from midlife onward. At the psychological level, feeling younger is associated with greater optimism, higher self-efficacy, and a more expansive future time perspective (Brothers et al., 2017). At the physiological level, Stephan et al. (2015) found that a younger subjective age was associated with lower C-reactive protein, a marker of systemic inflammation implicated in depression and cognitive decline. The same authors demonstrated that perceived age discrimination partially mediated the association between subjective age and biological aging.

Age identity captures the social-categorical dimension of subjective aging. Older adults who identify with a younger age group tend to report higher life satisfaction and better self-rated health, even after controlling for objective health status (Barrett, 2003). Spuling et al. (2013) used cross-lagged panel models to demonstrate bidirectional effects: subjective age predicted subsequent health, and health predicted subsequent subjective age, although the former pathway was stronger.

### 3.3 Behavioral mechanisms: health behaviors and social engagement

Levy and Myers (2004) demonstrated that older adults with more positive SPA were more likely to engage in preventive health behaviors—physical activity, balanced diet, medication adherence, regular check-ups—over a 20-year follow-up. Wurm et al. (2010) found that positive SPA predicted greater physical activity among middle-aged and older adults, with physical activity partially mediating the association between SPA and subsequent health.

Social engagement is equally important. Positive SPA predict greater social participation, larger and more diverse social networks, and greater likelihood of maintaining social roles (Cai et al., 2024). Social network diversity mediates the association between SPA and well-being (Meyer-Wyk and Wurm, 2024). Marital transitions shape SPA: widowhood is associated with declines, whereas remarriage with improvements (Turner et al., 2023).

### 3.4 Perceived control and goal processes

Perceived control over aging—domain-specific to the aging experience—predicts better physical and cognitive functioning, greater engagement in health-promoting behaviors, and higher psychological well-being (Lachman, 2006). According to the motivational theory of lifespan development (Heckhausen et al., 2010), the balance between primary and secondary control shifts across the lifespan, with late life necessitating greater reliance on secondary control.

Positive SPA expand the perceived opportunity structure for goal pursuit, while negative SPA constrict it (Dutt et al., 2018). Zhang and Neupert (2021) provided micro-longitudinal evidence: on days when older adults were more aware of age-related losses, they reported lower control beliefs and more negative affect; the reverse pattern emerged for days characterized by awareness of age-related gains. The SOC model further specifies that SOC strategies are more effective among those with positive SPA (Baltes and Baltes, 1990).

## 4 Sociocultural pathways and moderators

### 4.1 Ageism as a sociocultural antecedent of subjective aging

Ageism operates at multiple levels—institutional, interpersonal, and internalized—and constitutes a powerful sociocultural force shaping subjective aging constructs, especially SPA. The World Health Organization (2021b) identified ageism as a prevalent and pernicious form of discrimination. Chang et al. (2020) synthesized evidence from over 400 studies across 45 countries, finding that ageism was associated with significantly worse health outcomes, including a 7.5-year reduction in life expectancy attributable to negative aging self-perceptions originating from ageist cultural messages.

Kang and Kim (2022) found consistent evidence that experienced age discrimination, perceived ageism, and internalized age stereotypes were associated with increased depressive symptoms, greater anxiety, and lower life satisfaction, partially mediated by loneliness. A more recent study found that resilience buffered against the negative effects of ageism on mental health (Shen et al., 2025).

Internalized ageism, also referred to here as self-directed ageism, marks the transition from the sociocultural pathway to the perceptual pathway. It develops when ageist messages, discriminatory encounters, and institutional practices originating in the sociocultural environment are accepted and applied to the self. Once internalized, these beliefs become negative self-evaluations concerning one’s own aging and may therefore be expressed as a specific negative form of SPA. However, internalized ageism is not treated as synonymous with SPA: SPA is the broader and valence-neutral construct, whereas internalized ageism specifically denotes self-directed acceptance of devaluing or restrictive beliefs about older age. Using a syndemic framework, Christian and Halliday (2025) argued that self-directed ageism and depression may interact synergistically, with poverty, socioeconomic marginalization, gender inequality, and institutional neglect compounding mental health risks among older adults. At the institutional level, structural ageism in healthcare manifests as patronizing communication, exclusion from treatment decisions, and undertreatment of mental health conditions.

### 4.2 Cultural norms and cross-cultural variation

Löckenhoff et al. (2009) provided the most comprehensive cross-cultural investigation of aging perceptions across 26 cultures. They found that cultures placing higher value on older adults and maintaining stronger intergenerational ties held more positive views of aging. Research in Japan suggests that attitudes toward active aging are shaped by culturally embedded views of older adults as well as broader social and demographic conditions (Osawa et al., 2024).

Schönstein et al. (2023) compared AARC in Germany versus Burkina Faso and found that older adults in Burkina Faso reported higher awareness of age-related gains and lower awareness of age-related losses, despite objectively worse health. The authors attributed this to cultural integration: older adults in Burkina Faso are embedded in multigenerational households, maintain productive roles, and are viewed as sources of wisdom. These findings suggest that the association between objective health and SPA is culturally moderated and highlight the need for culturally sensitive interventions.

### 4.3 Intergenerational relationships and social networks

Positive intergenerational contact reduces negative age stereotypes and fosters more positive views of aging. A 2025 intervention using a “silent disco” event found that positive intergenerational contact reduced ageist attitudes and improved mood in both age groups, with effects persisting at one-month follow-up (Arenare et al., 2025). A 2025 systematic review found that intergenerational programs provided biopsychosocial benefits for older adults and supported active aging (Sánchez-Cazalla and Gutiérrez-Domingo, 2025).

Within families, the quality of relationships between older parents and adult children is associated with SPA. Perceived emotional support from adult children predicts more positive SPA, while perceived criticism or overprotection predicts more negative SPA. Marital quality independently predicts both SPA and well-being (Turner et al., 2023).

### 4.4 Socioeconomic and structural moderators

Socioeconomic status (SES) moderates the relationship between SPA and well-being. Economic inequality is associated with worse mental health in older adults, and this association is partly mediated by negative SPA (Lee and Allen, 2025). Gender differences in self-perceptions of aging appear to be domain-specific rather than uniform. Although men may report more positive attitudes toward age-related physical changes, women may report greater psychological growth, and some studies have found no overall gender difference in SPA (Bhattarai et al., 2025; Kim et al., 2021).

Personality traits represent individual-level moderators. Neuroticism is consistently associated with more negative SPA, while extraversion, openness, and conscientiousness predict more positive SPA (Carbone et al., 2024). Importantly, even after controlling for personality, SPA independently predict well-being outcomes, indicating incremental validity.

The digital divide—disparities in access to and proficiency with technology—may increasingly shape aging self-perceptions. Internet use has been found to reduce subjective age among older adults, primarily through improvements in health, self-efficacy, and social connection (Kong and Zhu, 2025). However, technology-related anxiety and perceived barriers can reinforce negative SPA and related subjective aging appraisals among those who struggle with digital tools.

**Figure 3:**
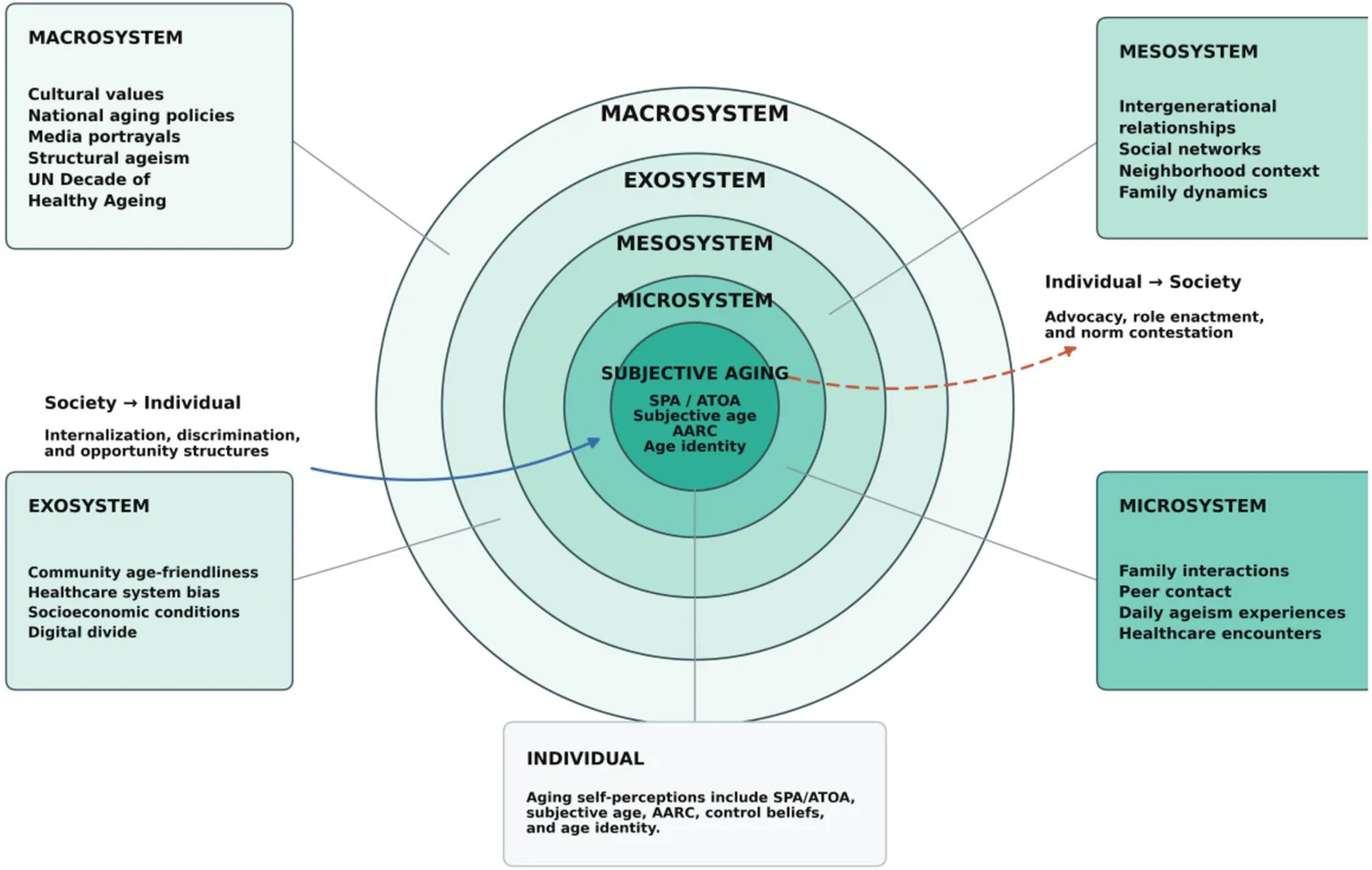
Multilevel ecological model of aging self-perceptions. Aging self-perceptions are situated within nested ecological systems (micro-, meso-, exo-, and macrosystem). These contexts shape how individuals perceive and experience aging, while individuals may also influence broader social environments.

Taken together, these sociocultural pathways and moderators suggest that subjective aging is not formed solely at the individual level, but is embedded within nested ecological systems, ranging from proximal interpersonal contexts to community, institutional, cultural, and policy environments. Figure 3 summarizes this ecological organization by locating aging self-perceptions across microsystem, mesosystem, exosystem, and macrosystem levels, while also indicating the potential for feedback between individual appraisals and broader social environments.

## 5 Psychological well-being outcomes

### 5.1 Depressive symptoms

The association between negative aging self-perceptions and depressive symptoms is among the most robust findings in this literature. Westerhof et al. (2023) found that negative subjective aging significantly predicted increased depressive symptoms across 107 longitudinal studies, with effects persisting after controlling for baseline depression, demographic variables, and physical health.

Multiple mechanisms account for this association. At the perceptual level, negative SPA promote hopelessness—a core feature of depression—by fostering the belief that age-related declines are inevitable, uncontrollable, and irreversible (Levy, 2022). At the behavioral level, negative SPA predict reduced engagement in activities that would otherwise provide reinforcement, consistent with behavioral models of depression (Lewinsohn, 1974). At the physiological level, construct-specific evidence has been reported for subjective age: a younger subjective age was associated with lower C-reactive protein levels (Stephan et al., 2015). This finding should not be interpreted as direct evidence concerning evaluative SPA.

The protective effect of positive SPA on depressive symptoms is particularly notable. Wurm and Schäfer (2022) found that gain-related SPA specifically predicted lower depressive symptoms and greater survival, suggesting that interventions targeting the cultivation of positive SPA—rather than merely reducing negative SPA—may confer unique benefits. Longitudinal evidence from the German Ageing Survey (DEAS) indicates that changes in SPA precede changes in depressive symptoms, supporting a directional pathway (Wurm et al., 2007).

### 5.2 Anxiety and death anxiety

Anxiety in later life—including generalized anxiety, health anxiety, and death anxiety—is also shaped by SPA and related subjective aging appraisals. More negative self-perceptions and attitudes toward aging are associated with greater death anxiety among older adults, with depressive symptoms serving as an additional independent predictor (Kulakçı Altıntaş and Ercan Şahin, 2025). The mechanism involves both cognitive (catastrophic interpretations of age-related bodily changes) and behavioral (avoidance of age-related information or healthcare) components.

Age-based stereotype threat has been shown to increase state anxiety in experimental settings (Lamont et al., 2015). A recent investigation found that the relationship between ageism, loneliness, and anxiety was moderated by resilience, suggesting that psychological resources can buffer the anxiety-provoking effects of negative SPA and related appraisals (Shen et al., 2025).

### 5.3 Life satisfaction and eudaimonic well-being

Positive SPA are associated not merely with the absence of distress but with the presence of flourishing. Older adults with positive SPA report higher life satisfaction, greater sense of purpose, ongoing personal growth, and more positive social relationships—the core dimensions of Ryff’s (1989) eudaimonic well-being. Bar-Tur (2021) argued that promoting positive SPA should be a central objective of interventions aimed at fostering late-life flourishing.

The distinction between hedonic and eudaimonic well-being is relevant. Positive SPA appear to promote both: they increase positive affect and life satisfaction (hedonic) while also enhancing meaning, purpose, and self-acceptance (eudaimonic). A 2025 study found that older adults with positive attitudes toward aging who also exhibited high flourishing reported the best health outcomes and illness recovery trajectories (Wu et al., 2025). This suggests a synergistic effect whereby positive SPA and eudaimonic well-being jointly confer resilience.

### 5.4 Loneliness and social well-being

Loneliness is a major global public health concern among aging populations. A 2025 systematic review and meta-analysis estimated that 27.6% of older adults worldwide experience loneliness, equivalent to more than one in four older adults (Salari et al., 2025). Negative SPA both predict and are predicted by loneliness, creating a reciprocal feedback loop. Older adults who view aging as a process of inevitable social loss are less likely to initiate and maintain social connections, which increases loneliness, which in turn reinforces negative perceptions of aging (Kang and Kim, 2022).

Conversely, positive SPA predict lower loneliness, greater social participation, and higher perceived social support (Cai et al., 2024). The social engagement pathway (Section 3.3) is critical: positive SPA motivate older adults to invest in social relationships, which provides a buffer against loneliness even during periods of objective social network contraction.

## 6 Empirical evidence across study designs

### 6.1 Longitudinal population-based studies

The strongest evidence for the association between subjective aging constructs and psychological well-being comes from large-scale longitudinal studies. The Ohio Longitudinal Study of Aging and Retirement (OLSAR)—the data source for Levy et al.’s (2002) landmark longevity finding—demonstrated that positive SPA predicted 7.5 years of additional survival over a 23-year follow-up. The German Ageing Survey (DEAS) has provided extensive evidence that SPA prospectively predict physical health, functional ability, and depressive symptoms across multiple waves (Wurm et al., 2007, 2013). The Health and Retirement Study (HRS) in the United States and the English Longitudinal Study of Ageing (ELSA) in the United Kingdom have enabled large-scale investigations of subjective age and health outcomes, consistently finding that a younger subjective age predicts better subsequent health (Stephan et al., 2018).

The ILSE study—a population-based cohort study in Germany—has been particularly informative for understanding SPA and cognitive outcomes. Siebert et al. (2018) demonstrated that negative ATOA at baseline predicted a significantly increased risk of being diagnosed with mild cognitive impairment or Alzheimer’s disease 12 years later, after controlling for relevant risk factors. The Baltimore Longitudinal Study of Aging (BLSA) contributed neuroimaging evidence: individuals with more negative views of aging showed a three-times-higher rate of hippocampal volume loss (Levy et al., 2016).

### 6.2 Experimental and priming studies

Experimental studies provide evidence for the causal influence of age stereotypes on outcomes relevant to psychological well-being. In a classic series of experiments, Levy (1996) demonstrated that subliminal priming of negative age stereotypes impaired memory performance and increased cardiovascular stress reactivity in older adults, while priming of positive age stereotypes produced opposite effects. These findings support the automatic, unconscious operation of stereotype embodiment (Levy, 2009).

Age-based stereotype threat experiments have shown that manipulating the salience of age stereotypes in testing situations impairs cognitive performance and increases anxiety (Lamont et al., 2015; Barber, 2017). Intervention studies using explicit stereotype disconfirming information have produced more mixed results, with some showing modest improvements in SPA and related subjective aging appraisals and others showing no significant change, highlighting the difficulty of modifying deeply internalized stereotypes through brief interventions alone.

### 6.3 Intervention studies

A growing number of intervention studies have targeted SPA as a modifiable risk factor. Wolff et al. (2014) demonstrated that adding a component targeting positive views on aging to a physical activity intervention enhanced both SPA and physical activity outcomes among older adults. Brothers and Diehl (2017) developed and tested the AgingPlus program, a multi-component intervention combining education about aging, cognitive reframing of age stereotypes, and behavioral activation. Participants showed significant improvements in SPA, physical activity, and psychological well-being at post-intervention and 8-week follow-up.

Educational interventions have also shown promise. Intergenerational contact interventions have also demonstrated beneficial effects on age-related stereotypes and older adults’ well-being. A 2025 systematic review found that intergenerational programs provided biopsychosocial benefits for older adults and supported active aging (Sánchez-Cazalla and Gutiérrez-Domingo, 2025).

### 6.4 Meta-analytic evidence

The updated meta-analysis by Westerhof et al. (2023) of 107 longitudinal studies provides the most comprehensive synthesis to date. The overall effect of subjective aging on health outcomes (including psychological well-being) was significant but small (LR = 1.347; 95% CI = 1.300–1.396), similar in magnitude to the earlier meta-analysis of 19 studies (Westerhof et al., 2014). Importantly, effects were stronger for multi-item measures of SPA than for single-item measures of subjective age, particularly for physical health outcomes. No significant moderation was found by chronological age, length of follow-up, type of health outcome, or study quality, indicating that the association is robust across methodological variations.

A 2025 meta-analysis of 33 studies found moderate associations between more positive self-perceptions of aging and better overall psychological well-being, including greater life satisfaction and lower levels of mental ill-health (Ugwu and Idemudia, 2025). Effect sizes were larger in studies that used multidimensional measures (such as AARC or AgeCog) compared to unidimensional measures (such as ATOA), supporting the value of distinguishing between gain- and loss-related perceptions.

To synthesize this heterogeneous evidence base, Table 2 summarizes representative empirical studies linking aging self-perceptions and related subjective aging constructs to psychological well-being outcomes. The table highlights differences in study design, measurement approach, key outcomes, proposed mediating mechanisms, moderators, and major limitations across longitudinal, intervention, and meta-analytic studies.

**Table 2:** Representative studies examining subjective aging constructs and psychological well-being or related health outcomes.

References	Study focus/Population	Study design/Data source	Subjective aging construct/measure	Key outcomes/Main findings	Main limitations
Levy et al. (2002)	Longevity: community-dwelling older adults (≥50 yrs., United States)	Longitudinal; 23-year follow-up; *N* = 660	ATOA subscale	Positive SPA associated with 7.5 years longer survival	Observational design; single baseline measure
Wurm and Schäfer (2022)	Mortality; German middle-aged and older adults	Longitudinal; 23 years; *N* ≈ 2,400; DEAS	AgeCog scales; subjective age	Gain-related SPA predicted lower mortality; loss-related SPA did not	Single-country sample; mechanisms not tested
Brothers et al. (2021)	Physical and mental health; adults aged ≥40 in the USA and Germany	Cross-national longitudinal study; 2.5-year follow-up	AARC-Gains and AARC-Losses	Personal aging perceptions linked age stereotypes to later health outcomes	Self-report data; limited assessment waves
Ugwu and Idemudia (2025)	SPA and psychological well-being; multiple countries	Meta-analysis; 33 studies; *N* = 43,556	Multiple SPA measures	Positive SPA moderately associated with better well-being; pooled r = 0.35	High heterogeneity; cross-sectional studies included
Westerhof et al. (2023)	Health and longevity; adults aged ≥50	Meta-analysis; 99 articles, 107 longitudinal studies	Multiple subjective aging measures	Favorable subjective aging predicted better health outcomes; LR = 1.347	Heterogeneous measures and outcomes; observational evidence
Siebert et al. (2018)	Cognitive impairment; German older adults	Longitudinal; 12-year follow-up; ILSE	ATOA subscale	Negative ATOA predicted greater risk of MCI or Alzheimer’s disease	Modest sample; observational design
Levy et al. (2016)	Alzheimer’s-related biomarkers; US older adults	Longitudinal neuroimaging study; BLSA	Negative age stereotypes	Negative stereotypes associated with faster hippocampal decline	Small subsamples; limited diversity
Kang and Kim (2022)	Ageism and psychological well-being; older adults	Systematic review; 13 studies	Experienced, perceived, and internalized ageism	Greater ageism associated with poorer psychological well-being	Mostly cross-sectional; varied measures
Schönstein et al. (2023)	Cross-cultural subjective aging; Burkina Faso and Germany	Cross-sectional comparative study	AARC-Gains and AARC-Losses	AARC patterns differed substantially across cultural contexts	Two-country comparison; self-report data
Zhang and Neupert (2021)	Daily aging experiences; US older adults	Micro-longitudinal repeated-measures study	State and trait awareness of aging	Control beliefs linked health fluctuations to aging awareness	Short observation period; self-report data
Stephan et al. (2015)	Subjective age and inflammation; US older adults	Cross-sectional; HRS; *N* = 4,120	Single-item subjective age	Younger subjective age associated with lower CRP	Cross-sectional design; single biomarker
Brothers and Diehl (2017)	AgingPlus intervention; US adults aged 50–82	Eight-week intervention; *N* = 62	Views on aging; perceived control	Preliminary improvements in aging views, control, and physical activity	Small sample; short follow-up

## 7 Risks, boundary conditions, and comparative analysis

### 7.1 Beyond the positive–negative dichotomy

The prevailing narrative in the SPA literature emphasizes the benefits of positive SPA and the harms of negative SPA. However, the positive–negative distinction may be more nuanced than it initially appears. Extremely positive views that minimize genuine age-related challenges could, in principle, reduce sensitivity to emerging health risks or make subsequent losses more difficult to accommodate. Direct empirical evidence for these potentially adverse effects remains limited, however, and positive SPA should not generally be interpreted as unrealistic denial. Future research should distinguish balanced positive appraisals from unrealistically optimistic views of aging.

Negative SPA may, in some contexts, serve adaptive functions. Defensive pessimism about aging could motivate greater engagement in preventive health behaviors. Acknowledgment of age-related limitations may facilitate adaptive goal adjustment and the use of compensatory strategies, consistent with the motivational theory of lifespan development (Heckhausen et al., 2010). The question is not simply “more positive is better” but rather “what type of aging self-perception, in what context, for which individual, leads to what outcome?”

### 7.2 Boundary conditions

The effects of subjective aging constructs on well-being vary across populations and contexts. Age itself moderates the association: the protective effect of a younger subjective age appears to strengthen with advancing chronological age (Pinquart and Wahl, 2021), consistent with the interpretation that it functions as an increasingly important self-enhancement strategy as age-related challenges accumulate. Health status is a critical moderator: among older adults with excellent objective health, SPA have relatively weaker associations with well-being (because well-being is already high), whereas among those with poor health, positive SPA function as a particularly important buffer (Wurm et al., 2013).

Personality traits moderate both the formation and consequences of SPA. Higher neuroticism is associated with more negative SPA and also amplifies their effects on depressive symptoms (O’Shea et al., 2017). Conversely, higher conscientiousness is associated with more positive SPA and may facilitate the behavioral translation of positive SPA into health-promoting actions.

### 7.3 Distinguishing subjective aging constructs and related psychological resources

Within the broader domain of subjective aging, SPA, subjective age, and AARC are correlated but non-equivalent constructs. SPA capture evaluative beliefs about one’s own aging; subjective age captures felt age and age-based identification; and AARC captures perceived gains and losses explicitly attributed to growing older. These constructs may differ in their antecedents, mechanisms, and associations with psychological well-being. The present review therefore does not treat one measure as a proxy for the entire subjective aging domain. Aging self-perceptions are conceptually related to but distinct from other psychological constructs relevant to late-life well-being. Generalized self-efficacy reflects a broad belief in one’s ability to handle challenges; SPA are domain-specific to the aging experience. Optimism is a generalized positive outcome expectancy; SPA are specifically about the aging process and may be more modifiable through aging-focused interventions. Resilience refers to the capacity to maintain functioning in the face of adversity; SPA may function as a resilience resource. Importantly, SPA demonstrate incremental validity: they predict well-being outcomes above and beyond these related constructs (Wurm et al., 2007) (Table 3).

**Table 3:** Comparison of major theoretical frameworks in subjective aging research.

Dimension	Stereotype embodiment theory (SET)	Motivational theory of lifespan development (MTLD)	Selection, Optimization, and Compensation (SOC) model	Awareness of age-related change (AARC) framework	Present multilevel framework
Core proposition/Theoretical focus	Age stereotypes internalized from culture become self-relevant in old age; operate implicitly through psychological, behavioral, and physiological pathways to shape health and well-being	Successful development requires flexible regulation of primary control (changing environment) and secondary control (adjusting self); control striving is a fundamental human motivation	Successful aging is achieved through three coordinated processes: selection of goals, optimization of resources for goal pursuit, and compensation for age-related losses; focus on “how” of aging well	Aging brings both gains (wisdom, emotional stability) and losses (physical decline, cognitive slowing); capturing adults’ awareness of these changes across five behavioral domains provides a multidimensional, process-oriented account of subjective aging	Subjective aging constructs influence psychological well-being through three interacting pathways (perceptual, behavioral, and sociocultural); integrates SET, MTLD, SOC, and AARC within a unified ecological framework.
Key constructs/Level of analysis	Age stereotypes (societal) → self-perceptions of aging (individual); stereotype threat; stereotype boost; implicit vs. explicit processing	Primary control (tenacious goal pursuit); secondary control (flexible goal adjustment, positive reappraisal); control capacity and control striving; developmental deadlines	Selection (elective, loss-based); Optimization (means, resources); Compensation (substitutive means); personal goals as organizing unit	AARC-gains and AARC-losses across five domains: health/physical, cognition, interpersonal, socioemotional, lifestyle/engagement	Perceptual pathway (stereotype embodiment, subjective age); behavioral pathway (health behaviors, social engagement); sociocultural pathway (ageism, cultural norms, intergenerational relations)
Mechanisms linking subjective aging constructs to well-being	Internalization: cultural stereotypes → self-definitions → self-fulfilling prophecies; operates via psychological (self-efficacy), behavioral (health behaviors), and physiological (stress reactivity) pathways	Goal engagement and disengagement: SPA shape perceived opportunity structure for goal pursuit; positive SPA expand opportunities, negative SPA constrict them	Adaptive resource allocation: SPA influence which goals are selected, how resources are optimized, and whether compensatory strategies are adopted; positive SPA facilitate SOC use	Perceptual awareness: greater awareness of gains → tenacious goal pursuit, intrinsic motivation; greater awareness of losses → flexible adjustment, compensatory strategies	Perceptual mediation (stereotype embodiment → interpretive bias → hopelessness/self-efficacy); behavioral mediation (SPA → health behaviors/social engagement → well-being); sociocultural mediation (ageism → internalized SPA → distress)
Empirical support/Key evidence	Longevity (Levy et al., 2002), cognitive pathology (Siebert et al., 2018), mediation by SPA (Brothers et al., 2021), experimental priming studies (Levy, 2009)	Longitudinal studies of goal adjustment and health (Wrosch et al., 2003); daily diary evidence of control belief mediation (Zhang and Neupert, 2021); SPA × control interactions (Dutt et al., 2018)	Cross-sectional and longitudinal associations between SOC strategy use and well-being; SOC moderated by SPA; domain-specific SOC-SPA interactions	Meta-analytic evidence (Westerhof et al., 2023); 23-year mortality prediction (Wurm and Schäfer, 2022); cross-cultural replication (Schönstein et al., 2023); micro-longitudinal (Zhang and Neupert, 2021)	Integrating evidence across SET, MTLD, SOC, and AARC traditions; supported by systematic reviews (Kang and Kim, 2022; Ugwu and Idemudia, 2025) and cross-cultural findings
Unique contributions	Explains how cultural-level age stereotypes manifest at individual level; explicit focus on unconscious operation; clear causal predictions from priming studies	Addresses the “how” of adaptive aging: not just whether SPA are positive, but how they facilitate or impede goal regulation; distinguishes primary from secondary pathways	Provides concrete behavioral strategies for successful aging; highlights agency and compensation; bridges individual psychology and behavioral implementation	Distinguishes gains vs. losses, overcoming limitations of unidimensional SPA measures; domain-specificity enables precise prediction; lifespan developmental grounding	First framework to explicitly integrate perceptual, behavioral, and sociocultural pathways; explicitly addresses bidirectional dynamics and ecological embedding; provides translational blueprint
Limitations/Open questions	Mediating pathways not fully specified; limited attention to positive stereotypes (stereotype boost); predominantly North American samples; cultural variation underexplored	Few direct tests of SPA–control–well-being sequences; limited integration with sociocultural context; personality moderation understudied	Normative assumptions about what constitutes “successful” aging; limited attention to cultural variation in what is selected/optimized; SOC as strategy vs. process distinction	Relatively recent construct; measurement standardization ongoing (AARC-SF validated but not yet universal); cross-cultural work only beginning	Requires empirical testing of integrated predictions; complexity may challenge parsimony; longitudinal multi-method designs needed; cultural adaptation of model untested
Implications for intervention	Modify age stereotypes through public education and media representation; target internalized stereotypes via cognitive reframing (AgingPlus program)	Promote flexible goal adjustment; teach primary and secondary control strategies; adapt goals to changing capacities	Teach SOC strategies in combination with SPA improvement; tailor goal selection to individual capacities and environmental resources	Distinguish gain vs. loss experiences in intervention design; build on perceived gains (not just reduce perceived losses); use AARC as process measure	Multi-level intervention architecture: individual (cognitive reframing, behavioral activation), community (intergenerational contact, age-friendly initiatives), policy (anti-ageism legislation, UN Decade of Healthy Ageing)
Reference(s)	Levy (2009, 2022), Brothers et al. (2021), Weiss and Kornadt (2018), Lamont et al. (2015)	Heckhausen et al. (2010), Wrosch et al. (2003), Dutt et al. (2018), Zhang and Neupert (2021)	Baltes and Baltes (1990), Zając-Lamparska (2021)	Diehl and Wahl (2010), Brothers et al. (2019), Kaspar et al. (2019), Wurm and Schäfer (2022), Schönstein et al. (2023)	Present review; Westerhof et al. (2023), Kang and Kim (2022), Chang et al. (2020), Ugwu and Idemudia (2025)

### 7.4 Research limitations

Several limitations characterize the existing evidence base. Measurement heterogeneity is pervasive: studies use different instruments (ATOA, AgeCog, AARC, subjective age items), making cross-study comparisons difficult. Sample selectivity remains a concern: most studies rely on relatively healthy, community-dwelling older adults in Western, high-income countries, limiting generalizability. Causal inference is constrained by the predominance of correlational designs; experimental and intervention studies remain relatively rare. Publication bias may inflate effect size estimates, although Westerhof et al. (2023) found no strong evidence of this. Finally, the field has been relatively slow to adopt standardized reporting practices, including preregistration, open data, and consistent use of validated outcome measures.

## 8 Translational implications

### 8.1 Individual-level interventions

The most direct translational implication of this literature is that SPA and related subjective aging appraisals are modifiable and that modifying them can improve psychological well-being. Cognitive reframing interventions target the perceptual pathway by helping older adults identify and challenge internalized negative age stereotypes, replacing them with more balanced, evidence-based views of aging. The AgingPlus program (Brothers and Diehl, 2017) demonstrated that an 8-week intervention combining psychoeducation about aging with cognitive restructuring techniques produced sustained improvements in SPA, physical activity, and well-being.

Behavioral activation approaches target the behavioral pathway by encouraging older adults to engage in activities that provide positive reinforcement, regardless of their existing SPA. Paradoxically, behavioral activation may also improve SPA: as older adults experience success and enjoyment through activity, their beliefs about what is possible in later life expand. Integrating behavioral activation with SPA-focused cognitive work may produce synergistic effects.

Psychoeducational interventions—providing accurate information about normative aging and challenging common myths—represent a low-intensity approach that could be disseminated widely through community organizations, healthcare settings, and digital platforms (Fuente-Hernández et al., 2025). A 2025 educational intervention trial with 140 older adults found significant improvements in both mental health and health-promoting behaviors following a structured program addressing aging knowledge and attitudes.

### 8.2 Community and intergenerational programs

Intergenerational contact interventions address the sociocultural pathway by creating opportunities for meaningful interaction between younger and older people. According to intergroup contact theory (Allport, 1954), contact reduces prejudice when it occurs under conditions of equal status, common goals, intergroup cooperation, and institutional support. A 2025 systematic review found that intergenerational programs generally produced psychosocial benefits across age groups, including improved well-being and social connectedness among older adults and reductions in negative age stereotypes among younger participants (Sánchez-Cazalla and Gutiérrez-Domingo, 2025).

Age-friendly community initiatives, guided by the WHO Age-Friendly Cities Framework, represent a population-level approach to modifying the environmental and social contexts that shape aging self-perceptions. By creating communities that value, include, and support older adults—through accessible public spaces, inclusive social programming, and anti-ageism campaigns—these initiatives aim to shift cultural norms about aging and, in turn, foster more positive aging self-perceptions at the individual level (WHO Age-Friendly Cities, 2021).

### 8.3 Policy and public health implications

At the policy level, the UN Decade of Healthy Ageing (2021–2030) provides a global framework for action that explicitly recognizes the importance of combating ageism and promoting positive perceptions of aging. Diehl et al. (2020) called for a “new narrative” of aging in society—one that acknowledges the realities of age-related change while emphasizing the potential for continued growth, contribution, and well-being. Such a narrative shift requires coordinated action across multiple sectors: media representation of older adults, healthcare provider training to reduce age bias, workplace policies that value older workers, and educational curricula that present accurate information about aging from childhood onward.

Healthcare systems can integrate SPA screening into routine care for older adults. Brief, validated measures (such as the AARC 10-item Short Form; Kaspar et al., 2019) could identify individuals with particularly negative SPA who may benefit from targeted intervention. Mental health services for older adults should explicitly assess and address SPA and related subjective aging appraisals as part of treatment planning, given their role in maintaining and exacerbating depressive and anxiety symptoms.

**Figure 4:**
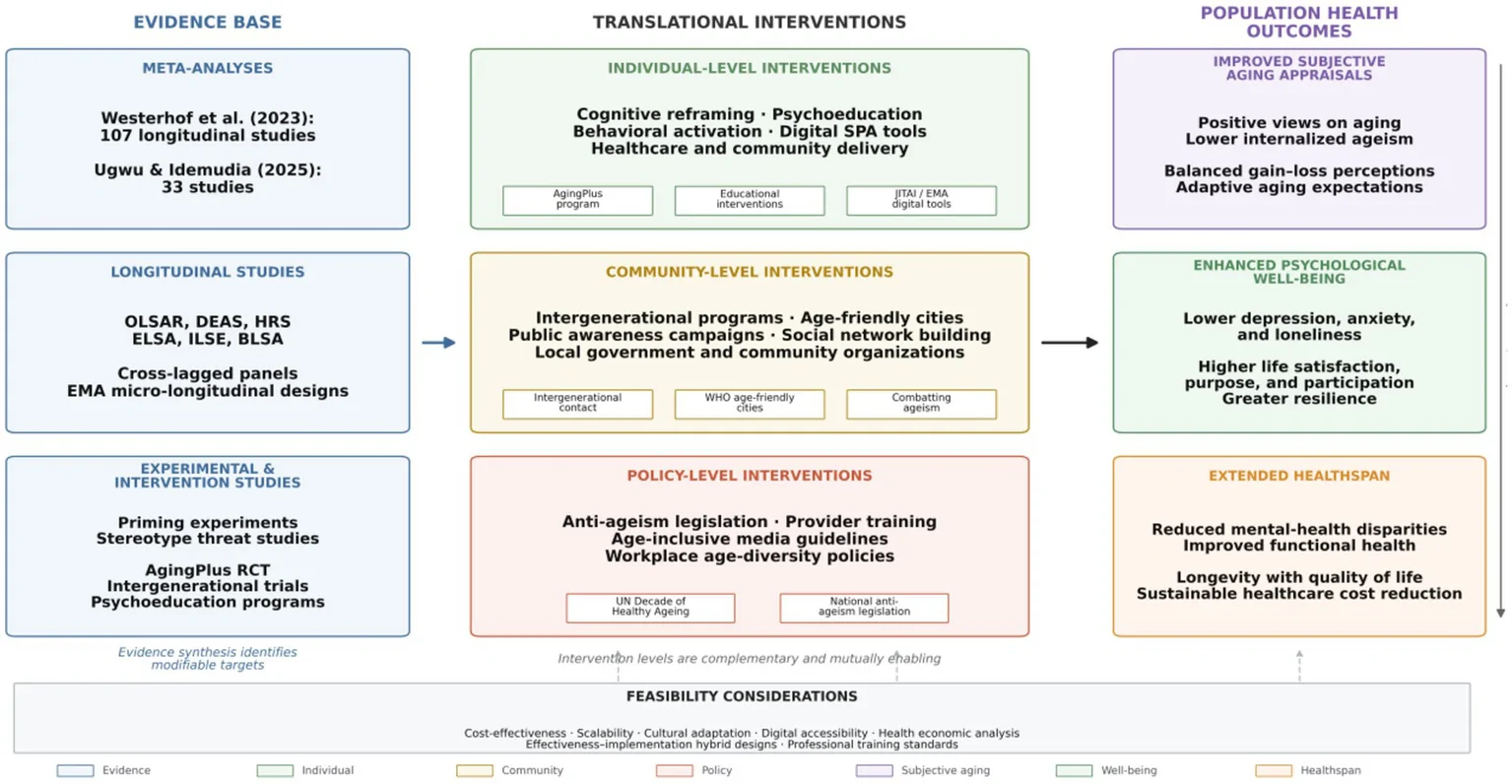
Translational ecosystem from subjective aging research to population health impact. Evidence from meta-analyses, longitudinal studies, and intervention research supports individual-, community-, and policy-level interventions, which improve subjective aging appraisals, psychological well-being, and healthspan. Feasibility factors condition their translation into population-level impact.

Overall, the translational value of subjective aging research lies in connecting evidence from longitudinal, experimental, and intervention studies to coordinated action across individual, community, and policy levels. Figure 4 summarizes this translational ecosystem, showing how evidence on aging self-perceptions can inform cognitive and behavioral interventions, intergenerational and age-friendly community programs, and broader public health strategies aimed at improving psychological well-being and healthy aging outcomes.

## 9 Emerging directions in subjective aging research

### 9.1 Digital technologies and subjective aging

The rapid digitalization of society—accelerated by the COVID-19 pandemic—has created new contexts for the formation and expression of aging self-perceptions. Internet use has been associated with younger subjective age among older adults, primarily through improved health, self-efficacy, and social connection (Kong and Zhu, 2025). Digital platforms may offer scalable channels for delivering SPA interventions. However, technology-related anxiety and perceived digital exclusion can reinforce negative aging self-perceptions among those who struggle to use digital tools (An et al., 2024).

Virtual reality (VR) and augmented reality (AR) technologies offer intriguing possibilities for SPA intervention. Immersive experiences that allow older adults to “inhabit” younger avatars or engage in activities that challenge age stereotypes could directly target the perceptual pathway. While research on VR-based SPA interventions is nascent, the broader literature on VR and older adult well-being suggests potential for novel, engaging approaches to aging self-perception modification.

### 9.2 Biomarkers and physiological correlates

The integration of biological markers into SPA research represents an important frontier. Stephan et al. (2015) demonstrated associations between subjective age and C-reactive protein, and Schönstein et al. (2022) reviewed emerging evidence linking SPA to objective biomarkers including cortisol, telomere length, and epigenetic aging clocks. These findings suggest that SPA and related subjective aging appraisals may “get under the skin” through stress-related physiological pathways, providing a mechanistic bridge between psychological experience and physical health. Future research should prioritize longitudinal investigations with repeated assessments of both SPA and biomarkers to establish temporal precedence and test mediational models.

### 9.3 Ecological momentary assessment and daily dynamics

Traditional SPA research has relied on global, retrospective self-report measures that cannot capture the dynamic, context-dependent nature of subjective aging appraisals. Ecological momentary assessment (EMA) methods—involving repeated, real-time assessments in naturalistic settings—are increasingly used to study the within-person dynamics of aging self-perceptions (Zhang and Neupert, 2021). EMA research has shown that awareness of aging fluctuates substantially within individuals across days and situations, and that these fluctuations predict same-day affect, control beliefs, and social behavior. This micro-longitudinal evidence complements traditional macro-longitudinal studies, revealing the proximal mechanisms through which aging self-perceptions influence daily well-being.

A 2024 daily diary study demonstrated considerable within-person variability in subjective age, with older adults feeling younger on days characterized by better mood, lower stress, and greater physical activity, highlighting the situationally embedded nature of subjective aging experiences (Schmidt et al., 2024). EMA methods also hold promise for intervention: just-in-time adaptive interventions could deliver brief SPA-focused content at moments when individuals are particularly aware of age-related losses, providing targeted support precisely when it is most needed.

## 10 Future directions and research agenda

### 10.1 Longitudinal and cross-lagged designs

Despite the substantial body of longitudinal evidence, causal inference remains limited. Most studies assess SPA at a single baseline and examine associations with subsequent outcomes, but far fewer employ cross-lagged panel designs that simultaneously model reciprocal effects. Spuling et al. (2013) demonstrated bidirectional associations between subjective age and health; similar designs should be applied to multidimensional SPA measures and psychological well-being outcomes. Long-term follow-ups (10+ years) with repeated assessments of both SPA and well-being are needed to establish the temporal dynamics and durability of effects.

### 10.2 Mechanistic precision and causal testing

Mediational analyses in the SPA literature have been predominantly cross-sectional or involve only two time points, which cannot establish temporal precedence of mediators. Future research should adopt designs with at least three time points and employ modern causal mediation methods to rigorously test the perceptual, behavioral, and sociocultural pathways proposed in the present framework. Experimental manipulation of specific pathway components—such as random assignment to conditions that differentially target stereotype internalization versus behavioral activation—would provide stronger evidence for causal mechanisms.

### 10.3 Cross-cultural validation

The vast majority of SPA research has been conducted in Western, high-income contexts. Cross-cultural differences in the content, formation, and consequences of subjective aging constructs remain poorly understood. Multisite cross-cultural studies with harmonized measures are needed to establish the generalizability of findings and identify culturally specific moderators. Participatory co-design approaches—involving older adults from diverse cultural backgrounds in the development of SPA measures and interventions—would enhance ecological validity and cultural appropriateness (Schönstein et al., 2023).

### 10.4 Standardized outcome metrics

The heterogeneity of outcome measures in SPA research limits cumulative knowledge development. Westerhof et al. (2023) called for minimum reporting standards including validated symptom measures (e.g., PHQ-9, GAD-7), process-based constructs (e.g., rumination, behavioral activation), and session-level engagement metrics. For interventions, the inclusion of both SPA-specific measures (e.g., AARC Short Form) and well-being outcomes would facilitate comparison across studies. Preregistration, open data, and harmonized data formats are essential for advancing the field.

### 10.5 Interdisciplinary framework development

Advancing this field requires integration across clinical psychology, social gerontology, health psychology, cultural psychology, and public health. The multilevel framework proposed here represents one step toward such integration, but further theoretical refinement is needed. Hybrid effectiveness–implementation study designs would enable simultaneous evaluation of efficacy, feasibility, and scalability. Health economic analyses should be incorporated to inform cost-effectiveness and potential reimbursement strategies. Professional training standards for clinicians working with older adults should include content on subjective aging constructs and their role in mental health.

## 11 Conclusion

The evidence reviewed here demonstrates that subjective aging—how individuals experience, interpret, and evaluate their own aging—is not merely a reflection of objective circumstances but an active psychological domain that shapes trajectories of psychological well-being. Throughout this review, subjective aging has been treated as an umbrella domain comprising related but non-equivalent constructs, including SPA, subjective age, AARC, and age identity.

Key findings warrant emphasis. First, the meta-analytic evidence is now robust: Westerhof et al. (2023) confirmed across 107 longitudinal studies that subjective aging significantly predicts health and well-being outcomes, with effects that are small in magnitude but consistent across diverse populations and designs. Second, multidimensional measures of SPA that distinguish between age-related gains and losses provide more precise prediction than unidimensional measures, highlighting the importance of assessing not only the absence of negative perceptions but the presence of positive ones (Wurm and Schäfer, 2022). Third, ageism—operating at institutional, interpersonal, and internalized levels—functions as a social determinant of aging self-perceptions, and combating ageism is therefore a public health priority (Chang et al., 2020; Kang and Kim, 2022). Fourth, SPA and related subjective aging appraisals are modifiable: intervention studies have demonstrated that cognitive reframing, behavioral activation, and intergenerational contact can improve SPA and, through SPA, psychological well-being.

Clinically, these findings suggest that mental health professionals working with older adults should routinely assess SPA and related subjective aging appraisals as part of case formulation and treatment planning. Brief validated instruments, such as the AARC Short Form (Kaspar et al., 2019), can efficiently identify individuals whose negative aging self-perceptions may be contributing to psychological distress. Interventions that target aging self-perceptions—either as a standalone approach or as an adjunct to evidence-based treatments such as CBT—hold promise for enhancing outcomes and addressing the substantial treatment gap in late-life mental health.

The translation from research to practice requires attention to the boundary conditions identified in this review. Not all positive SPA are adaptive in all contexts; interventions should promote realistic, balanced views of aging rather than simplistic positivity. Cultural sensitivity is essential: approaches developed in Western contexts must be adapted for other settings through participatory co-design with local stakeholders. The digital divide must be addressed to ensure equitable access to technology-mediated interventions.

Looking ahead, the field requires rigorous longitudinal designs that establish temporal precedence, experimental studies that isolate causal mechanisms, cross-cultural research that establishes generalizability, and standardized measurement that enables cumulative knowledge development. The UN Decade of Healthy Ageing provides both a policy framework and a moral imperative for this work. By advancing understanding of how aging self-perceptions shape psychological well-being—and by developing evidence-based strategies to foster adaptive aging self-perceptions—researchers and clinicians can contribute to a future in which longer lives are also lives of greater psychological richness, meaning, and well-being.
